# Comparing treatment intensification and clinical outcomes of metformin and dipeptidyl peptidase‐4 inhibitors in treatment‐naïve patients with type 2 diabetes in Japan

**DOI:** 10.1111/jdi.13088

**Published:** 2019-06-21

**Authors:** Takeshi Horii, Makiko Iwasawa, Jyunichi Shimizu, Koichiro Atsuda

**Affiliations:** ^1^ Pharmacy Practice and Science I, Research and Education Center for Clinical Pharmacy Kitasato University School of Pharmacy Sagamihara Kanagawa Japan; ^2^ Research and Education Center for Clinical Pharmacy Division of Drug Information Kitasato University School of Pharmacy Sagamihara Kanagawa Japan; ^3^ Department of Pharmacy Tokyo Saiseikai Central Hospital Tokyo Japan

**Keywords:** Oral antidiabetic drugs, Treatment intensification, Type 2 diabetes mellitus

## Abstract

Japan's guidelines emphasize tailored therapy, but do not guide physicians on the use of a specific regimen in drug‐naive patients. The role of long‐term initial therapy could be important in key elements of diabetes treatment, such as continuation of the initially prescribed drug. We investigated the frequency of occurrence to treatment intensification after the initiation of metformin or dipeptidyl peptidase‐4 inhibitor treatment. In multivariable‐adjusted Cox proportional hazards models, initiation of dipeptidyl peptidase‐4 inhibitor was associated with a low hazard of intensification. The findings of this survey showed that dipeptidyl peptidase‐4 inhibitors were the preferred first‐line treatment in Japan because of the high continuation rate of the treatment and hemoglobin A1c‐lowering effect. This information would provide guidance in selecting initial hypoglycemic drugs to optimize the treatment of type 2 diabetes mellitus patients in Japan and Asia.

## Graphical abstract

We investigated the role of the initial therapy in long‐term treatment by examining the time to treatment intensification after commencement of metformin or dipeptidyl peptidase‐4 inhibitor.

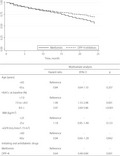

## Introduction

Although metformin is recommended as first‐line therapy in the American Diabetes Association/European Association for the Study of Diabetes consensus recommendations, ∼40% of patients initiating oral antidiabetic drugs (OADs) have been reported to have not received this recommended initial therapy^1^, ^2^, ^3^. Furthermore, the applicability of these recommendations to Asians, including the Japanese, has not been clarified. Compared with Caucasians, type 2 diabetes mellitus in Asians tends to be characterized more by impaired insulin secretion than by increased insulin resistance. Therefore, the choice of OADs depends on patient factors, such as age, complications and risk of hypoglycemia, while considering the characteristics and side‐effects of each drug^4^, ^5^.

According to a study that investigated the prescription trend of initial OADs selection for type 2 diabetes mellitus patients in Japan, dipeptidyl peptidase‐4 inhibitors (DPP‐4I) were the most preferred first‐line OADs, followed by metformin^6^. When selecting a hypoglycemic agent, not only medical outcomes, but also patients’ quality of life and attitude toward treatment, should be considered. The frequency of treatment intensification affects the quality of life of type 2 diabetes mellitus patients. Therefore, it is important for the initial OADs not only to have good glycemic control impact, but also to allow patients to continue the initial treatment. In the present study, we investigated the frequency of occurrence to treatment intensification and the effect of blood glucose control at the end of a 2‐year observation in treatment‐naïve patients who were first treated with metformin or DPP‐4I.

## Methods

A retrospective cohort study was carried out at Tokyo Saiseikai Central Hospital in Tokyo, Japan. We included patients who newly initiated either metformin or DPP‐4I between January 2009 and March 2015. The exclusion criteria were: (i) type 1 diabetes; (ii) age <18 years; (iii) an estimated glomerular filtration rate <30 mL/min/1.73 m^2^; and (iv) a follow‐up period <2 years. We reviewed medical records to retrieve the patients’ clinical information.

The primary outcome was the frequency of occurrence to treatment intensification, defined as initiating or adding another class of OADs (include glucagon‐like peptide 1 agonists) or insulin. The secondary outcome was hemoglobin A1c (HbA1c) levels after a 2‐years observation period.

Normally distributed numerical data are presented as the mean ± standard deviation. Categorical variables were analyzed using Fisher's exact test and the χ^2^‐test, and are expressed as absolute numbers or percentages. Continuous variables were analyzed using an unpaired Student's *t*‐test. To evaluate the association of the frequency of occurrence to treatment intensification, Kaplan–Meier statistics were used and assessed using the Cox regression analysis, which was adjusted for age, sex, estimated glomerular filtration rate, body mass index and baseline HbA1c, and expressed as a hazard ratio and 95% confidence interval. We used patient characteristics at baseline as the predictor variables in univariate analysis, carried out multivariate analysis using factors found to be *P* < 0.2 and calculated the odds ratio.

Differences were regarded as significant when *P* < 0.05. All statistical analyses were carried out using the Stata software (version 10; StataCorp, College Station, TX, USA). The present study was carried out in accordance with ethical guidelines for medical and health research involving human participants. The ethics board of the Tokyo Saiseikai Central Hospital approved the study (control number: 27–11).

## Results

The 930 patients who met the inclusion criteria (Table 1) consisted of 624 (67.0%) and 306 patients (33.0%) who initiated metformin and DPP‐4I, respectively. Compared with initiation of DPP‐4I, patients who initiated metformin were younger, more likely to be obese and less likely to have chronic kidney disease.

**Table 1:** Patient characteristics

	Mean ± SD or *n* (%)			*P* (metformin vs DPP‐4I)
	Overall *n* = 930	Metformin *n* = 624	DPP‐4I *n* = 306	
Male, *n* (%)	711 (76.5)	411 (76.7)	233 (76.1)	0.877
Age (years)	60.1 ± 11.3	58.1 ± 10.6	64.5 ± 11.5	
<65, *n* (%)	310 (33.3)	157 (25.2)	153 (50.0)	<0.001
65≤, *n* (%)	620 (66.7)	467 (74.8)	153 (50.0)	
HbA1c (%)	7.9 ± 2.0	7.9 ± 2.0	8.0 ± 1.9	
<7.0, *n* (%)	177 (19.0)	120 (19.2)	57 (18.6)	0.384
7.0 to <8.0, *n* (%)	372 (40.0)	247 (39.6)	125 (40.9)	
8.0, *n* (%)	381 (41.0)	257 (41.2)	124 (40.5)	
BMI (kg/m^2^)	25.1 ± 8.7	25.8 ± 9.9	23.5 ± 4.2	
<25, *n* (%)	564 (60.6)	336 (53.8)	228 (74.5)	0.004
25≤, *n* (%)	366 (39.4)	288 (46.2)	78 (25.5)	
eGFR (mL/min/1.73 m^2^)	73.3 ± 16.9	75.1 ± 15.6	69.7 ± 18.6	
<60, *n* (%)	177 (19.0)	91 (14.6)	86 (28.1)	<0.001
60≤, *n* (%)	753 (81.0)	533 (85.4)	220 (71.9)	
Diabetes duration (years)	4.9 ± 5.9	5.0 ± 5.8	4.7 ± 6.3	0.461

Data are presented as the mean ± standard deviation. BMI, body mass index; eGFR, estimated glomerular filtration rate; HbA1c, hemoglobin A1c; SD, standard deviation.

### Treatment intensification

**Figure 1:**
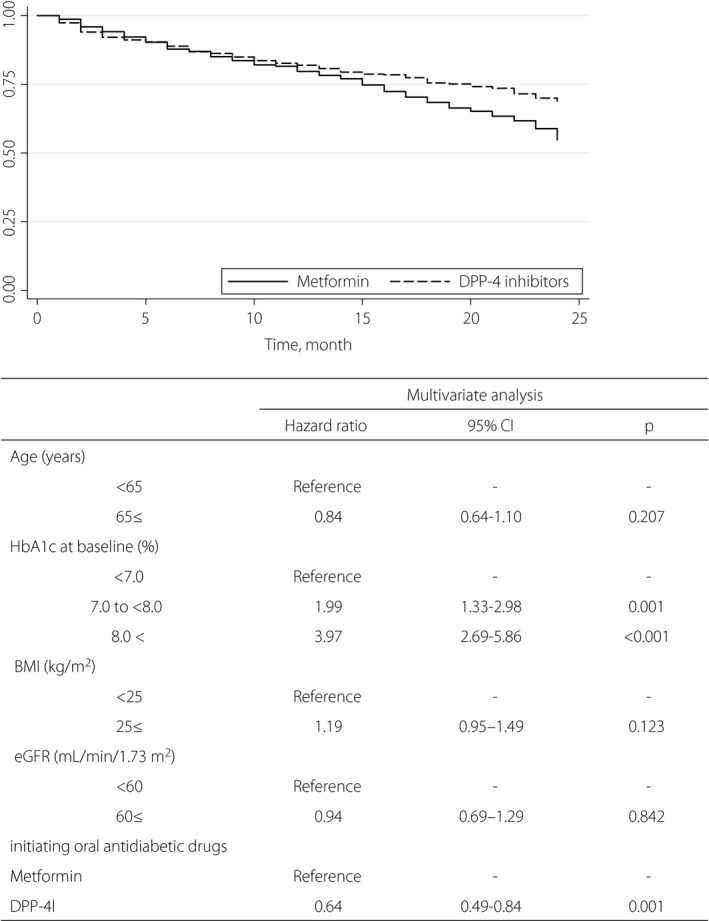
Rates of treatment intensification were significantly lower for dipeptidyl peptidase‐4 inhibitor (DPP‐4I; 31.0%) than for metformin (45.2%), respectively (log–rank test, *P*  < 0 .001). Statistical comparison of treatment intensification during 24 months in patients with type 2 diabetes mellitus with the initiation of metformin or DPP‐4I treatment is shown in the inserted table. Hazard ratio is the Cox proportional hazards model adjusted by sex, age, hemoglobin A1c (HbA1c), body mass index (BMI) and estimated glomerular filtration rate (eGFR). CI, confidence interval.

Compared with the initial period of 12 months, the rate of treatment intensification in the latter 12 months was significantly higher for both metformin (initial 12 months vs latter 12 months: 20.4% vs 56.7%, *P* < 0.001) and DPP‐4 (initial 12 months vs latter 12 months: 18.0% vs 37.8%, *P* < 0.001). These results were also significant in the multivariable Cox proportional hazards models: HbA1c value of 7.0% to <8.0%, >8.0% and initiation of DPP‐4I (Figure 1). The transition of HbA1c between 6 and 12 months after the start of observation (6 months, 12 months, 18 months, 24 months) was 6.91 ± 0.78, 7.02 ± 1.01, 6.92 ± 0.74 and 7.06 ± 0.88 for DPP‐4I, and 7.29 ± 0.93, 7.34 ± 0.97, 7.30 ± 0.93 and 7.42 ± 0.97 for metformin, respectively. The results of the multivariate analysis adjusted (Table 2) showed that the following were statistically significant: HbA1c value of 7.0 to <8.0%, >8.0%, body mass index ≥25 (kg/m^2^) and initiation of DPP‐4I.

**Table 2:** Logistic regression model for factors related to the achievement of hemoglobin A1c value of <7.0% at the end of the 2‐year of follow‐up observation period at baseline characteristics

	Multivariate analysis		
	Odds ratio	95% CI	*P*
Age (years)			
<65	Reference	–	–
65	1.35	0.97–1.88	0.083
HbA1c at baseline (%)			
<7.0	Reference	–	–
7.0 to <8.0	0.32	0.21–0.49	<0.001
8.0	0.27	0.17–0.41	<0.001
BMI (kg/m^2^)			
<25	Reference	–	–
25	0.64	0.47–0.87	0.005
eGFR (mL/min/1.73 m^2^)			
<60	Reference	–	–
60	0.96	0.65–1.42	0.842
Initiating oral antidiabetic drugs			
Metformin	Reference	–	–
DPP‐4I	1.94	1.39–2.72	<0.001

Adjusted Age, hemoglobin A1c (HbA1c) at baseline, body mass index (BMI), estimated glomerular filtration rate (eGFR), initiating oral antidiabetic drugs. CI, confidence interval; DPP‐4I, dipeptidyl peptidase‐4 inhibitors.

## Discussion

We showed that patients initially prescribed DPP‐4I were significantly less likely to require treatment intensification than those prescribed metformin. These observations for DPP‐4I were presumed to be attributable to less frequent administration, similar impact on weight gain^7^, ^8^, ^9^, ^10^, ^11^, ^12^, ^13^ and enhanced tolerance compared with metformin^14^, ^15^. Treatment intensification could be undesirable to patients^16^, ^17^, ^18^, and inherently associated with increased adverse effects, costs and complexity of medical treatment.

The present study showed that DPP‐4I displayed an advantage in achieving an HbA1c value <7.0% compared with metformin. A previous study has reported that DPP‐4Is were associated with a smaller decline in HbA1c and a lower chance of reaching the HbA1c goal of <7% than metformin, suggesting the inferiority of DPP‐4I to metformin as monotherapy^19^. However, previous studies have suggested that incretin‐based therapies, such as DPP‐4I, were more effective in Asian patients than they were in Caucasian patients^20^, ^21^, ^22^, ^23^, ^24^. Furthermore, a cohort study in Japan has shown that the glucose‐lowering effects of DPP‐4I and metformin are similar^25^. However, in patients with a high value of HbA1c (approximately 8%), DPP‐4I is more effective compared with metformin at the onset of OAD^25^. In the present study, DPP‐4I had a higher odds ratio for achieving HbA1c >7% than metformin did, because the baseline HbA1c was relatively high, at approximately 8%. DPP‐4I was superior to metformin in lowering HbA1c, which could translate into reduced need for treatment intensification. Furthermore, transition of HbA1c tended to rise from 6 to 12 months in the case of both metformin and DPP‐4I. The HbA1c in DPP‐4I tended to be closer to the target value of Japan's glycemic control of 7.0%, whereas HbA1c transitioned at approximately 7.3% in the case of metformin at 6 months or later. For these reasons, the rate of achieving the HbA1c target with metformin is estimated to be low compared with DPP‐4I. Therefore, it can be considered that the rate of treatment intensification was high at 12 months or later.

The present study had some limitations that are worth mentioning. First, the decision to make treatment intensification from initial choice of OADs had no clear criteria and was at the discretion of the physician. Therefore, it could not be ruled out that the particular physician's treatment policy might have influenced the outcome. Second, the present study was retrospective; thus, we could not examine detailed reasons for the treatment intensification, such as lack of therapeutic effect, side‐effects and cost.

In conclusion, these findings showed that DPP‐4I was one of the preferred first‐line treatments in Japan because of a high rate of treatment continuation and superior HbA1c‐lowering effect. This information would provide guidance in selecting initial OADs to optimize type 2 diabetes mellitus management in Japan, as well as the rest of Asia. Maintaining initial treatment and achieving HbA1c targets might reduce the likelihood of diabetic complications and improve quality of life, so details need to be investigated.

## Disclosure

The authors declare no conflict of interest.

## References

[1] Desai NR , Shrank WH , Fischer MA , *et al* Patterns of medication initiation in newly diagnosed diabetes mellitus: quality and cost implications. Am J Med Mar 2012; 125: 302.e1–7. 10.1016/j.amjmed.2011.07.033 PMC4347833 22340932

[2] Rafaniello C , Arcoraci V , Ferrajolo C , *et al* Trends in the prescription of antidiabetic medications from 2009 to 2012 in a general practice of Southern Italy: a population‐based study. Diabetes Res Clin Pract 2015; 108: 157–163. 25686508 10.1016/j.diabres.2014.12.007

[3] Berkowitz SA , Krumme AA , Avorn J , *et al* Initial choice of oral glucose lowering medication for diabetes mellitus: a patient‐centered comparative effectiveness study. JAMA Intern Med 2014; 174: 1955–1962. 25347323 10.1001/jamainternmed.2014.5294

[4] The Japan Diabetes Society . Treatment Guide for Diabetes 2014–2015. Available from http://www.jds.or.jp/modules/en/index.php?content_xml:id=1#guide Accessed August 7, 2017.

[5] Treatment Guide for Diabetes 2016–2017. Edited by Japan Diabetes Society. Bunkodo Co.

[6] Murayama H , Imai K , Odawara M . Factors influencing the prescribing preferences of physicians for drug‐naive patients with type 2 diabetes mellitus in the real‐world setting in Japan: insight from a Web Survey. Diabetes Ther 2018; 9: 1185–1199. 29696567 10.1007/s13300-018-0431-3 PMC5984934

[7] Garber AJ . Novel incretin‐based agents and practical regimens to meet needs and treatment goals of patients with type 2 diabetes mellitus. J Am Osteopath Assoc 2011; 111: 20–30. 21813733

[8] Charbonnel B , Karasik A , Liu J , *et al* Efficacy and safety of the dipeptidyl peptidase‐4 inhibitor sitagliptin added to ongoing metformin therapy in patients with type 2 diabetes inadequately controlled with metformin alone. Diabetes Care 2006; 29: 2638–2643. 17130197 10.2337/dc06-0706

[9] Rosenstock J , Sankoh S , List JF . Glucose‐lowering activity of the dipeptidyl peptidase‐4 inhibitor saxagliptin in drug‐naive patients with type 2 diabetes. Diabetes Obes Metab 2008; 10: 376–386. 18355324 10.1111/j.1463-1326.2008.00876.x

[10] Rosenstock J , Aguilar‐Salinas C , Klein E , *et al* Effect of saxagliptin monotherapy in treatment‐naive patients with type 2 diabetes. Curr Med Res Opin 2009; 25: 2401–2411. 19650754 10.1185/03007990903178735

[11] Aschner P , Kipnes MS , Lunceford JK , *et al* Effect of the dipeptidyl peptidase‐4 inhibitor sitagliptin as monotherapy on glycemic control in patients with type 2 diabetes. Diabetes Care 2006; 29: 2632–2637. 17130196 10.2337/dc06-0703

[12] Raz I , Hanefeld M , Xu L , *et al* Efficacy and safety of the dipeptidyl peptidase‐4 inhibitor sitagliptin as monotherapy in patients with type 2 diabetes mellitus. Diabetologia 2006; 49: 2564–2571. 17001471 10.1007/s00125-006-0416-z

[13] Nauck MA , Meininger G , Sheng D , *et al* Efficacy and safety of the dipeptidyl peptidase‐4 inhibitor, sitagliptin, compared with the sulfonylurea, glipizide, in patients with type 2 diabetes inadequately controlled on metformin alone: a randomized, double‐blind, non‐inferiority trial. Diabetes Obes Metab 2007; 9: 194–205. 17300595 10.1111/j.1463-1326.2006.00704.x

[14] Thomas MC , Paldánius PM , Ayyagari R , *et al* Systematic Literature Review of DPP‐4 Inhibitors in patients with type 2 diabetes mellitus and renal impairment. Diabetes Ther 2016; 7: 439–454. 27502495 10.1007/s13300-016-0189-4 PMC5014795

[15] Wu S , Chai S , Yang J , *et al* Gastrointestinal adverse events of dipeptidyl peptidase 4 inhibitors in type 2 diabetes: a systematic review and network meta‐analysis. Clin Ther 2017; 39: 1780–1789. 28827024 10.1016/j.clinthera.2017.07.036

[16] Grant RW , Pabon‐Nau L , Ross KM , *et al* Diabetes oral medication initiation and intensification: patient views compared with current treatment guidelines. Diabetes Educ 2011; 37: 78–84. 21115980 10.1177/0145721710388427 PMC3033981

[17] Huang ES , Brown SE , Ewigman BG , *et al* Patient perceptions of quality of life with diabetes‐related complications and treatments. Diabetes Care 2007; 30: 2478–2483. 17623824 10.2337/dc07-0499. PMC2288662

[18] Peyrot M , Rubin RR , Lauritzen T , *et al* Resistance to insulin therapy among patients and providers: results of the cross‐national Diabetes Attitudes, Wishes, and Needs (DAWN) study. Diabetes Care 2005; 28: 2673–2679. 16249538 10.2337/diacare.28.11.2673

[19] Karagiannis T , Paschos P , Paletas K , *et al* Dipeptidyl peptidase‐4 inhibitors for treatment of type 2 diabetes mellitus in the clinical setting: systematic review and meta‐analysis. BMJ 2012; 344: 1–15. 10.1136/bmj.e1369 22411919

[20] Amori RE , Lau J , Pittas AG . Efficacy and safety of incretin therapy in type 2 diabetes: systematic review and meta‐analysis. JAMA 2007; 298: 194–206. 17622601 10.1001/jama.298.2.194

[21] Yang W , Pan CY , Tou C , *et al* Efficacy and safety of saxagliptin added to metformin in Asian people with type 2 diabetes mellitus: a randomized controlled trial. Diabetes Res Clin Pract 2011; 94: 217–224. 21871686 10.1016/j.diabres.2011.07.035

[22] Seino Y , Fujita T , Hiroi S , *et al* Efficacy and safety of alogliptin in Japanese patients with type 2 diabetes mellitus: a randomized, double‐blind, dose‐ranging comparison with placebo, followed by a long‐term extension study. Curr Med Res Opin 2011; 27: 1781–1792. 21806314 10.1185/03007995.2011.599371

[23] Seino Y , Rasmussen MF , Nishida T , *et al* Efficacy and safety of the once‐daily human GLP‐1 analogue, liraglutide, vs glibenclamide monotherapy in Japanese patients with type 2 diabetes. Curr Med Res Opin 2010; 26: 1013–1022. 20199137 10.1185/03007991003672551

[24] Cai X , Han X , Luo Y , *et al* Efficacy of dipeptidyl‐peptidase‐4 inhibitors and impact on β‐cell function in Asian and Caucasian type 2 diabetes mellitus patients: a meta‐analysis. J Diabetes 2015; 7: 347–359. 25043156 10.1111/1753-0407.12196

[25] Azuma K , Yasunori S , Koichi K , *et al* Relationship between the efficacy of oral antidiabetic drugs and clinical features in type 2 diabetic patients (JDDM38). J Diabetes Investig 2016; 7: 386–395. 10.1111/jdi.12430 PMC4847894 27330726

